# Optimizing cognitive interventions to improve real-world function for healthy older adults

**DOI:** 10.1007/s10433-025-00852-2

**Published:** 2025-03-24

**Authors:** Rachel Wu, Tania M. Rodriguez, Bethany P. Tavenner, Isadora Farias Lopes de Queiroz, Walter Boot, Jeanine Parisi, Michelle Carlson, Martin Lövdén, Margaret E. Beier, Alan Gow

**Affiliations:** 1 Department of Psychology, University of California, Psychology Building, 900 University Avenue, Riverside, CA 92521 USA; 2 Division of Geriatrics and Palliative Medicine, Weill Cornell Medicine, New York City, USA; 3 Department of Mental Health, Johns Hopkins Bloomberg School of Public Health, Baltimore, USA; 4 Department of Mental Health, Johns Hopkins Bloomberg School of Public Health and Johns Hopkins Center On Aging and Health, Johns Hopkins University, Baltimore, USA; 5 Department of Psychology, University of Gothenburg, Gothenburg, Sweden; 6 Department of Psychological Sciences, Rice University, Houston, USA; 7 Department of Psychology, Heriot-Watt University, Edinburgh, UK

**Keywords:** Cognitive interventions, Real-world function, Functional decline, Cognitive aging

## Abstract

Healthy aging requires acquiring new functional skills for adaptation in a dynamic environment. Cognitive interventions with older adults have largely focused on improving broad cognitive abilities, aiming for transfer to functional effects. By contrast, interventions focusing directly on acquiring new functional skills can address current real-world issues, including the need for reskilling and reducing the digital divide, especially for underserved communities. In doing so, we may better understand how aspects of age-related learning and cognitive and functional decline may be due to suboptimal learning circumstances rather than senescence. In this opinion paper, we highlight key aspects for designing long-lasting, real-world interventions to improve functional skills, and potentially transfer to cognitive effects, for older adults. This approach could help build more inclusive theories of cognitive aging, while progressing the field toward developing more effective and useful interventions.

## Introduction

Over the past several decades, cognitive interventions with healthy older adults have been shown to enhance cognitive abilities via exercising those abilities (see Hertzog et al. 2008). The ultimate goal of these interventions is to translate the observed cognitive benefits into real-world functional gains. This expectation is reasonable given that functional skills use cognitive abilities, and that the decline of both are related (Tucker-Drob 2011). Despite promising interventions showing that cognitive change is possible, few cognitive interventions have translated into long-term, real-world functional benefits (Owen et al. 2010; Simons et al. 2016). However, such outcomes may not have been targeted, as the focus on function typically occurs only after basic functions start declining (e.g., getting dressed, managing money, taking medications, Lawton and Brody 1969; WHO 2021). Healthy adults, who by definition have intact basic functional abilities, may still have difficulties performing new tasks in daily life, such as learning to use a new smartphone or updated software. As such, there is an urgent need for interventions that focus on acquiring real-world skills to adapt to changes in environmental, occupational, and personal demands (e.g., Lövdén et al. 2010; Nguyen et al. 2020).

This paper first briefly discusses cognitive abilities versus functional skills in relation to cognitive interventions. Then, we highlight real-world problems currently faced by many older adults, including the need for reskilling and the digital divide. We explain the benefits of shifting the emphasis from increasing cognitive abilities to increasing functional skills (with increasing cognitive abilities as a potential secondary benefit) in healthy older adults. We then highlight key aspects for designing long-lasting, effective real-world cognitive interventions for older adults, including aspects of behavior change research. Finally, we provide recommendations for future research, including community-based participatory research approaches.

### Cognitive abilities versus functional skills in relation to cognitive interventions

Cognitive interventions (unimodal or multimodal—engaging in single or multiple activities and/or training single or multiple abilities) largely fall into two categories: cognitive training and cognitive engagement. Training cognitive abilities, such as working memory can improve those abilities, with some evidence of slight transfer to other untrained abilities, especially when training multiple abilities simultaneously (Butler et al. 2018; Gajewski et al. 2020; Rosenberg et al. 2018; Schmiedek et al. 2010; see Melby-Lervag et al. 2016; Lampit et al. 2014; Soveri et al. 2017 for reviews). Cognitive training interventions typically provide participants with tasks that exercise a specific cognitive ability, such as working memory (e.g., via remembering the location of objects on a computer screen). By contrast, cognitive engagement interventions asking participants to learn real-world skills (e.g., photography) can exercise particular cognitive abilities, such as episodic memory (e.g., a list of words as a proxy of remembering specific details of past events; Leanos et al. 2023; Park et al. 2014). Cognitive engagement interventions typically have focused on increasing cognitive abilities as the outcome (e.g., Carlson et al. 2008; Leanos et al. 2023; Park et al. 2014), and therefore, the impact on functional skills is less clear. In contrast to cognitive abilities, functional skills can be defined as experience-dependent skills (i.e., reliant on environmental input, see Greenough et al. 1987). For example, there is no genetic program for becoming proficient at using a new smartphone or playing the piano, though learning and executing these skills require broad cognitive abilities (e.g., working memory, processing speed).

Traditional cognitive training approaches that include increasing specific cognitive abilities could provide the cognitive resources to support the ability to learn new skills to foster adaptation, and cognitive training could in principle occur alongside functional training. However, for busy adults who have limited time to spend on improving their abilities and limited funding for research on cognitive aging, now is the time to prioritize real-world function (as opposed to cognitive abilities) as the main outcome to support independent living. For example, the ability to remember the digits 48,596 backwards is moot if someone cannot arrange a doctor’s appointment because that has to be booked through an online portal and the older adult is not able to do so. The next section highlights two important examples of this point.

### Real-world issues: two examples

Besides learning for fun (e.g., hobbies, leisure activities), many healthy older adults may face situations that require them to learn new skills and/or information to foster positive aging experiences. For example, some older adults may need to learn new software or procedures to maintain their job. Others may need to update technological devices when older ones become obsolete to maintain contact with others. Some older adults move closer to their adult children and need to learn how to complete tasks in a new place. Many older adults also need to adapt to new health issues that may require them to learn new routines (e.g., learning to administer shots, learning to use a non-dominant hand). Older adults also can be forced into new roles, such as becoming a caregiver for an elderly family member, learning to live in a new residence if they need to move to a more manageable home, and learning how to manage many things solo if they become widowed.

Two prominent examples of adapting to new or changing environments include reskilling/upskilling due to working later in life (e.g., Beier et al. 2020) and low proficiency with current technological devices and software (e.g., Charness and Boot 2022). In terms of reskilling/upskilling, employees often have specific skill sets suited for their roles. Yet, the dynamic nature of the workplace, especially considering technological advancements, requires employees to continue to both adapt existing skills and acquire new skills. This challenge of skill development (reskilling and upskilling) is particularly necessary for older adults who are staying in full-time work positions longer than previous generations (e.g., Beier et al. 2020). Most executives list new skill learning for employees as a high priority, especially because the types of jobs that are available are changing (Illanes et al. 2018). Other reports estimate that by 2025 half of employees worldwide will need reskilling/upskilling to remain in their positions (Schwab and Zahidi 2020). As the percentage of older adults who are employed full-time increases, companies will continue to require their workers to adapt to changing needs with increasing focus on older adult learning. Adult learning opportunities tend to be self-directed and informal (i.e., unstructured; Beier et al. 2017), leaving it up to the individual to acquire new skills to remain in the workforce (Lund et al. 2021). Very little is known about how to support novel skill learning and adaptation in adult workers. The fact that there is an increasing number of older adults who need or want to stay in the workforce is important to consider alongside the fact that it is difficult for them to find new employment after losing their job (Wanberg et al. 2016). The facilitation of acquiring relevant skills may in turn facilitate the ability for older adults to remain in the workforce, benefiting both the individual and society (e.g., Beier et al. 2020).

The digital divide is responsible for a large portion of reskilling/upskilling issues that relate to keeping up with technological advances, such as learning to use new technological devices and navigating new online features or software. The digital divide refers to the reduced or lack of digital/technology access, literacy, or usage (e.g., computers, Internet) among certain individuals in society, and it is often based on demographic characteristics (e.g., age, socioeconomic status, residential area, etc.; Smith 2014; Van Dijk 2005; Vogels 2021). In contrast to younger adults, older adults are specifically affected by the digital divide, as they are less likely to use or adopt technology as a result of barriers they encounter (e.g., physical health issues such as decreased eyesight, low confidence; Charness and Boot 2022; Czaja et al. 2006; Smith 2014; Vaportzis et al. 2017). Older workers must remain up to speed with the technological advances occurring in their work environment to stay employed or to find a new job (Colbert et al. 2016; Wallace 2004). Moreover, being skillful with technology is also important as it is increasingly being implemented by many service providers for things such as booking medical appointments, ordering food at restaurants, monitoring one’s bank account, accessing benefits portals, and ordering transportation or grocery services (e.g., Bate et al. 2021; Suntai and Beltran 2023). Therefore, technology training can significantly improve functional skills for older adults with or without cognitive impairment in a variety of domains (Czaja et al. 2024; Harvey et al. 2021).

It is important to note that the issues of reskilling/upskilling and the digital divide are particularly prominent in underserved communities. For example, the need for reskilling/upskilling may disproportionately affect older adults who cannot retire because they lack the resources to do so and/or are not eligible for social services (e.g., Ayón et al. 2023). In addition, lower socioeconomic status and being a racial/ethnic minority contribute to the digital divide (Fang et al. 2019; Mossberger et al. 2003). A study assessing Internet use patterns among low-income older adults showed that those categorized as never or discontinued users were more likely to have the lowest income levels and more likely to be Black or Hispanic (Choi and DiNitto 2013). Another study also identified that the intersectionality of having low socioeconomic status and being a racial/ethnic minority contributed to the digital divide (Yoon et al. 2020). Suntai and Beltran (2023) found that females that were Black and Hispanic had lower access to technology than White females and Black, Hispanic, and White males. Other studies have pointed out the influence that cultural gender roles may have on technology access and usage. For example, Hispanic women have been found to have more technology access limitations than Hispanic men due to having less power over financial means (Casado-Muñoz et al. 2015; Orellano-Colón et al. 2024). Underserved older adults might face unique access barriers that are specifically relevant to one or more of their socially disadvantaged identities (e.g., cost if they are low-income; Cresci 2010; Graham 2010; Yazdani-Darki et al. 2020). These studies highlight how older adults with underserved backgrounds are disproportionately more affected by the digital divide (Fang et al. 2019; Mossberger et al. 2003), which then could have implications for reskilling and upskilling, much of which depends on technological advances (e.g., Autor 2024). Future research on functional interventions for older adults should strengthen the ability of older adults to adapt and increase their skill sets in both work and personal contexts for diverse populations.

### Shifting focus from cognitive to functional for healthy older adults

Given the need for more research on facilitating skill learning in older adulthood, we encourage research inquiry to refocus cognitive interventions from targeting cognitive abilities to investigating ways to best support skill learning/enhancement. This shift would prioritize the overall goal of maintaining independent living, which is linked to the WHO definition of healthy aging (WHO 2021), while filling critical knowledge gaps in cognitive aging research. In terms of benefiting healthy older adults, this shift would matter most for busy adults who have limited time to spend on improving their abilities, especially underserved older adults who may be less able to choose when to retire (e.g., Ayón et al. 2023) and/or have strong demands on their time and energy, such as caregiving obligations (e.g., del-Pino-Casado et al. 2018). Importantly, this shift would address immediate, ongoing needs, including reskilling/upskilling and improving technology proficiency described above. As such, research findings on improving skill learning would be able to better prepare older adults for everyday challenges.

In terms of scientific benefits, the shift would provide testable hypotheses that, if followed through, would improve our understanding of how much of age-related cognitive decline is due to a suboptimal learning environment rather than senescence. This shift would build on and go beyond the existing literature on lifelong learning and the benefits of cognitive engagement/stimulation in older adulthood (e.g., Hertzog et al. 2008; Park et al. 2014; Schaie and Willis 2010; Stine-Morrow et al. 2007) by identifying older adults’ specific functional skill needs and developing tailored interventions addressing those needs. From the cognitive engagement and neuroplasticity literatures, we now know that positive change is possible in older adulthood, and that change is most likely to occur from multi-domain, real-world, meaningful experiences (e.g., Gavelin et al. 2021; Krzeczkowska et al. 2021). Aging theories also have incorporated the possibility of gains (e.g., selection, optimization, and compensation theory, Baltes 1997; The Scaffolding Theory of Aging and Cognition, Reuter-Lorenz and Park 2014). Perhaps we can go even further to discover that what is currently thought of as normative cognitive decline through senescence because only so much can be changed could actually be partially due to situational factors related to unaddressed learning needs (see Stine-Morrow and Manavbasi 2022). Resulting changes to existing theories and newly developed theories may then be more ecologically valid and inclusive. Once the sources of cognitive and functional decline are determined, then the field can progress toward developing long-lasting, effective interventions for older adults (and perhaps preventive interventions for younger and middle-aged adults), as opposed to addressing symptoms without addressing the underlying cause.

Inspiration for the refocusing of cognitive interventions, and cognitive aging research more broadly, can be drawn from research earlier in the lifespan, when children and adolescents typically experience long-term, real-world functional gains. In these younger populations, functional skills are a priority (e.g., reading, math), and functional and cognitive abilities increase in tandem in the natural environment without necessarily training cognitive abilities in isolation (Wu et al., in press). Therefore, for older adults, especially those with less time and resources, instead of training cognitive abilities, it may be more advantageous to teach specific skills useful for daily life, with increasing broad cognitive abilities (related to knowledge and functional skills) as a secondary outcome. To be fair, the debate over whether it is more useful to train specific skills or broad cognitive abilities for learners, especially younger learners, spans over a century (see Katz et al. 2018).

### Key aspects of long-lasting real-world cognitive intervention design

This section presents key aspects for designing long-lasting, real-world cognitive interventions for older adults, including aspects from behavior change research (e.g., Duckworth and Gross 2020; Hughes et al. 2023; Klusmann et al. 2021; Nielsen et al. 2018). In particular, two key aspects are: (1) identifying and addressing individual barriers and facilitators; and (2) providing support in the interventions to mitigate the barriers and promote the facilitators. These barriers and facilitators are important to identify and either mitigate or promote by the individual and others because they determine what we attend to, how we think about situations, and how we respond (Duckworth and Gross 2020). Lengthier discussions of how to change behavior in the service of healthy aging in general have been published elsewhere (e.g., Klusmann et al. 2021), but some aspects related to novel skill learning are highlighted below.

#### Identifying barriers and facilitators of learning

Surveys and interviews have been conducted to learn about what older individuals may encounter as barriers or facilitators to maintaining cognitive abilities (e.g., Mehegan et al. 2017; Niechcial et al. 2023; Vaportzis and Gow 2018; Vaportzis et al. 2017), which may be related to barriers and facilitators for learning for adaptation. There are individual (e.g., prior experiences, motivation, physical health, individual resources such as time and money), social (e.g., family/friend support), environmental (e.g., safety), and societal (e.g., community resources, stereotypes, learning opportunities) barriers and facilitators. Learning new skills often requires time, money, transportation, health, energy, awareness of available learning opportunities, instructors, learning materials, and self-efficacy/learning motivation (e.g., Kanfer and Ackerman 2004; Mehegan et al. 2017; Rodriguez et al. 2022; Vaportzis and Gow 2018). All of these components highlight that learning is a privilege that may not be afforded to every older adult (Wu et al. 2021). Some barriers are more easily modified or induced (e.g., not knowing about learning opportunities), whereas others may be more difficult or impossible to modify (e.g., chronic fatigue). Likewise, some facilitators are more easily induced, such as an accessible list of low-cost or free learning opportunities, compared to others, such as services that free up an older adult caretaker’s time and energy to be able to take new classes.

Motivational barriers related to both the individual and the activities have been discussed in the literature in relation to older adults’ activity choices. For example, some older adults may prioritize short-term advantages and short-term well-being over long-term advantages and long-term well-being (e.g., Carstensen 1999; Hess 2014), which is linked to future time perspective (i.e., perceived time left in life, Teuscher and Mitchell 2011). Some older adults may prioritize long-term advantages or learn for the sake of learning, in which case high motivation could be a facilitator for novel skill learning for adaptation. Relatedly, another motivational barrier/facilitator is perceived effort needed to perform a learning activity relative to the utility of the activity (e.g., Kanfer and Ackerman 2004), and perceived effort can be related to self-efficacy and prior experiences. Whether an activity provides purpose or has a clear functional outcome is also important for older adults (e.g., interacting with grandchildren more, can help save money or time, Vaportzis et al. 2017). Performance goals (i.e., goals based on overall capabilities) might be prioritized rather than learning goals (i.e., goals based on learning progress), especially if an older adult holds a fixed mindset (i.e., believing that abilities cannot improve with effort) rather than a growth mindset (Dweck 2006; Klusmann et al. 2021). Older adults’ prior experiences in educational environments also affect their motivation to engage in future learning (Gorges and Kandler 2012). Although some groups hold positive aging beliefs, many still believe that older adults cannot learn new skills and that decline is inevitable (Niechcial et al. 2023). All of these motivational factors can affect persistence and goal striving (i.e., the path to attaining a goal) in terms of activity engagement (e.g., Brinkhof et al. 2023).

#### Providing support in the interventions to mitigate the barriers and promote the facilitators

After barriers and facilitators have been identified, learning interventions can then implement methods to reduce the barriers while promoting facilitators where feasible. For example, a community of low-income Latino older adults may face language and financial barriers (e.g., Rodriguez et al. 2022), prompting the intervention to be conducted in Spanish and to provide free learning resources to increase learning engagement. Barriers also could vary across community members, requiring tailored assistance, such as addressing specific health or cognitive needs. Checking on participants throughout the intervention and offering flexibility as barriers emerge within an intervention would help prevent attrition.

One potentially effective way of recruiting and retaining older participants in a learning intervention could be telling them about the importance of learning new skills for adaptability, thereby encouraging purpose and motivation (e.g., Goldberg and Kiernan 2005). Interventions could first demonstrate short-term gains by focusing on current learning needs to then convey messages about long-term gains. Alternatively, interventions could convey the need to set aside short-term well-being for long-term gains when learning new difficult skills. Interventions also could convey the cost of not learning or engaging in a particular task, such as losing future opportunities or economic resources. However, interventions should also convey risks in engaging in a particular task, such as increased potential for scams with increased engagement with technology.

In order to maintain the effects of learning interventions long-term, encouraging persistence by providing support for coping with avoidance, challenges, and setbacks could be considered and implemented. For example, open discussions on these topics, as well as peer accountability, have been useful in prior learning interventions by increasing both interest and engagement (Leanos et al. 2023). In general, motivation has been found to be enhanced in settings where older adults are in a group while learning (National Research Council 2012); socialization is a commonly reported facilitator supporting intervention participation (e.g., Niechcial et al. 2023). Scaffolded approaches in terms of instructional support (e.g., tip cards, cheat sheets, Sanders et al. 2024) also could be useful to help older adults gain skills at an appropriate pace, similar to Vygotsky’s notion of *zone of proximal development* in child development (e.g., Vygotsky 1978). These supports would recognize the unique needs and abilities of older adults (Czaja and Sharit 2012).

### Implications for future research

Focusing more on function supports the needs for community-based participatory research that would address real-world urgent issues, while also promoting inclusivity. There are vast individual differences in older adults in terms of baseline cognitive abilities, functional skills, cultural background, motivations, etc. Despite the heterogeneity, future research could determine common factors among clusters of older adults to develop tailored interventions based on various barriers and intersections of different barriers, as well as identifying the most urgent skills to include first in interventions for different communities. Very little is known about how to best teach real-world skills to diverse older adults. For example, how might aspects of andragogy and pedagogy interact for completely novel skills? How might intergenerational or peer interactions facilitate or hinder learning for older adults? Could interventions use a foot-in-the-door approach to motivate older adults who resist learning novel skills? Future research could test different ways of motivating older adults to engage in novel skill learning to produce long-term cognitive and functional benefits (see Hess 2014).

This research area also should develop new measures to assess real-world functional gains and adaptability. At the moment, functional measures (e.g., IADLs) have a very low bar and involve minimal to no learning (see Nguyen et al. 2020). Better learning and adaptability measures are needed that directly relate to current real-world tasks, perhaps based on the newly learned skills (e.g., learning to use a new online banking platform, Czaja et al. 2017; Harvey et al. 2020). These measures also would provide an assessment for adaptability to new real-world learning tasks. Adaptability (also referred to as flexibility) measures for the most part are currently simplified cognitive tasks, as seen in standard IQ tests, as opposed to more complex real-world situations (see Stine-Morrow and Manavbasi 2022). Solid measures would then allow for more inquiry into knock-on effects of novel skill learning on mental and physical health, purpose, and potentially even life satisfaction (e.g., Martinez et al. 2024). However, these measures would be difficult to implement and may rapidly become obsolete due to technological advancements. Therefore, these measures would need to be constantly updated to most accurately reflect current functional abilities.

After developing measures to assess real-world functional gains and adaptability, future research could potentially tease apart sources of functional and/or learning decline in healthy older adults. In particular, research could disentangle cases of decline based on modifiable versus non-modifiable factors. For example, two potentially modifiable factors could be: (1) long-term engagement in mostly habitual tasks (i.e., a situational deficit from not practicing learning how to learn and relevant abilities for an extended period), and (2) mindset and motivational issues (e.g., holding negative stereotypes). These potentially modifiable factors would contrast with other factors that would be more difficult or impossible to modify, such as unrecoverable neural deficits.

Finally, more research could be conducted on how early development and education can better prepare adults to continue adapting via novel skill learning throughout their lifespan. Previously acquired knowledge and skills provide a basis for understanding new tasks and for the development of learning strategies. The learning history (including formal and informal learning) is thus a critical factor for predicting and explaining individual differences in novel skill learning. Early schooling experiences and related childhood conditions (e.g., interactions with parents and peers) are also likely to set the stage for motivations and expectations related to future learning. Thus, preparing our children for lifelong learning may be as important as designing intervention for improving functional skills in later life. Furthermore, connecting cognitive aging research with child cognitive development research would provide a fruitful lifespan approach (see Wu et al. 2017), given the overlapping research questions including how to increase cognitive and functional abilities and learning motivation, while avoiding stagnation/decline.

## Conclusions

In summary, cognitive interventions have focused on improving cognitive abilities, with the ultimate goal of translating the cognitive improvements into real-world functional skills. This paper proposes that cognitive interventions focusing more on functional skills than on cognitive abilities as direct outcomes with healthy older adults can have several important benefits for the individual, society, and the cognitive aging field. Individual/societal benefits include addressing current real-world issues, such as reskilling/upskilling and the digital divide, especially for underserved communities. We would also better understand how suboptimal learning environments (as opposed to senescence) lead to age-related functional and/or learning decline, while developing more inclusive theories and more effective and useful cognitive interventions. We suggest learning interventions identify and address facilitators and barriers to learning, leveraging research on behavior change, such as conveying the importance of learning new skills and focusing on necessary skills first, as well as providing ways to help cope with and accept challenges, setbacks, and avoidance. Future cognitive intervention research can develop more tailored approaches based on individual learning facilitators and barriers. Moreover, future research can develop more ecologically valid measures for functional gains and adaptability, while also better understanding sources of functional/learning decline in healthy older adults, and across the rest of the lifespan. More traditional cognitive training approaches could provide the cognitive resources to support the ability to learn new skills that are necessary to adapt, but this paper proposes that the primary outcome for cognitive interventions should be real-world function to support independent living in a changing environment. The question is no longer about whether we should engage in new learning to remain cognitively healthy, but rather about the best ways to learn new skills and information given that we have to do so to adapt. Ultimately, these efforts would allow researchers to optimize cognitive interventions to potentially help older adults live happier, well-adjusted lives.

## Data Availability

No datasets were generated or analyzed during the current study.
